# Supplementary figures and images for: Quantitative trait loci and transcriptome signatures associated with avian heritable resistance to Campylobacter
Source: Sci Rep. 2021 Jan 12;11:1623. doi: 10.1038/s41598-020-79005-7 (PMC7804197; doi:10.1038/s41598-020-79005-7)

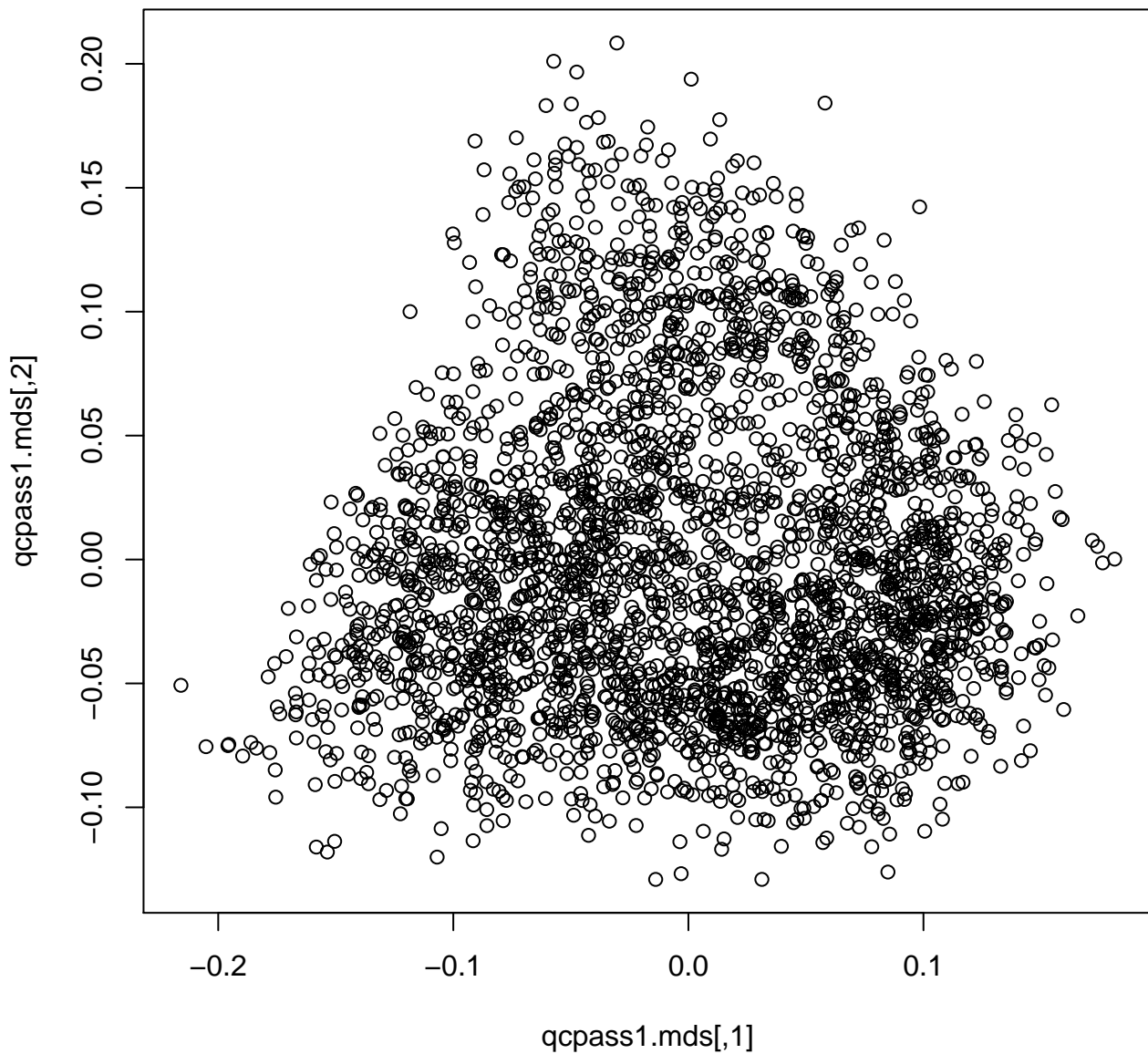

Supplement: Supplementary file 1 — Supplementary Figure S1. [file 41598_2020_79005_MOESM1_ESM.pdf]

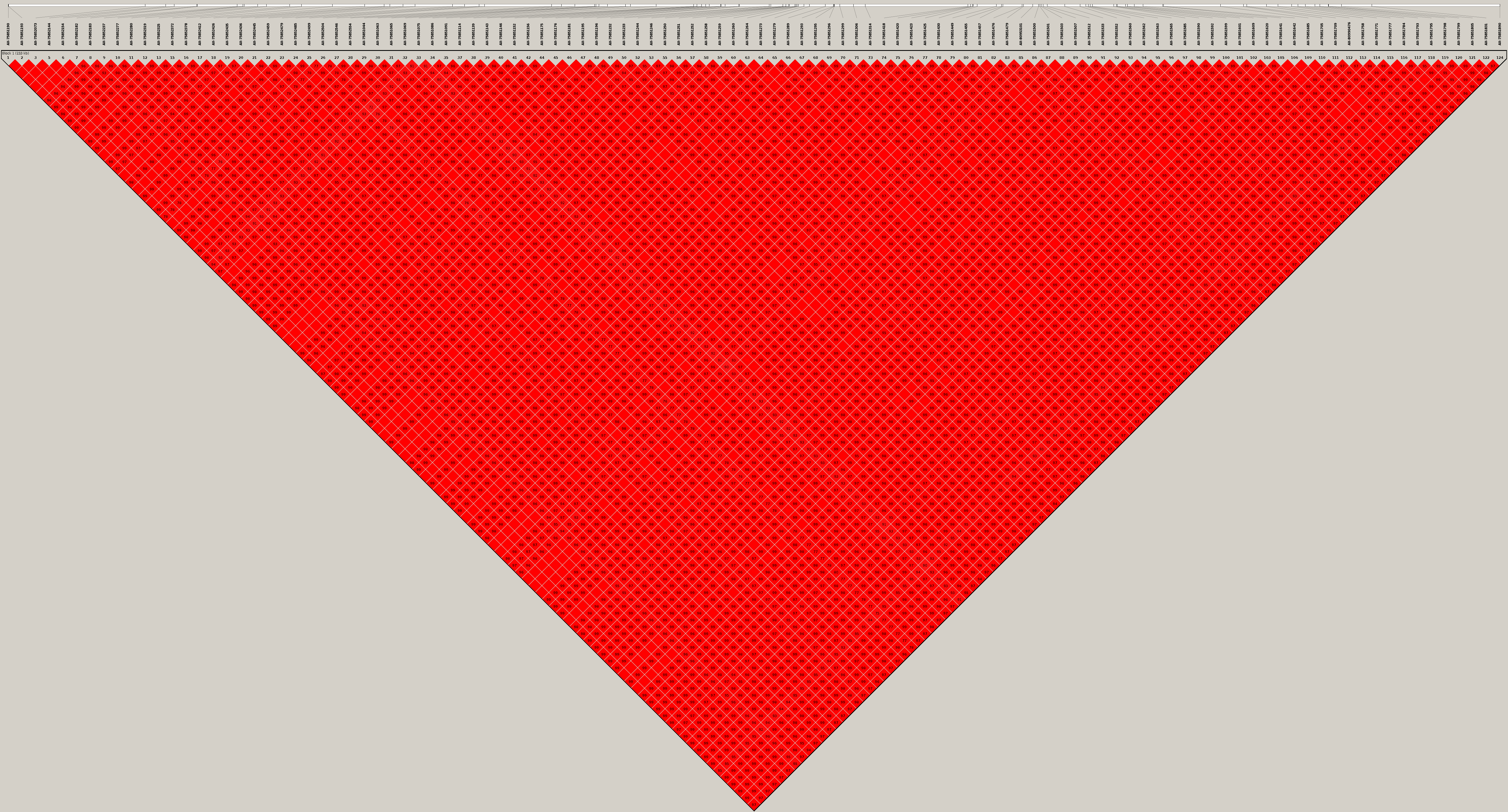

Supplement: Supplementary file 2 — Supplementary Figure S2. [file 41598_2020_79005_MOESM2_ESM.png]

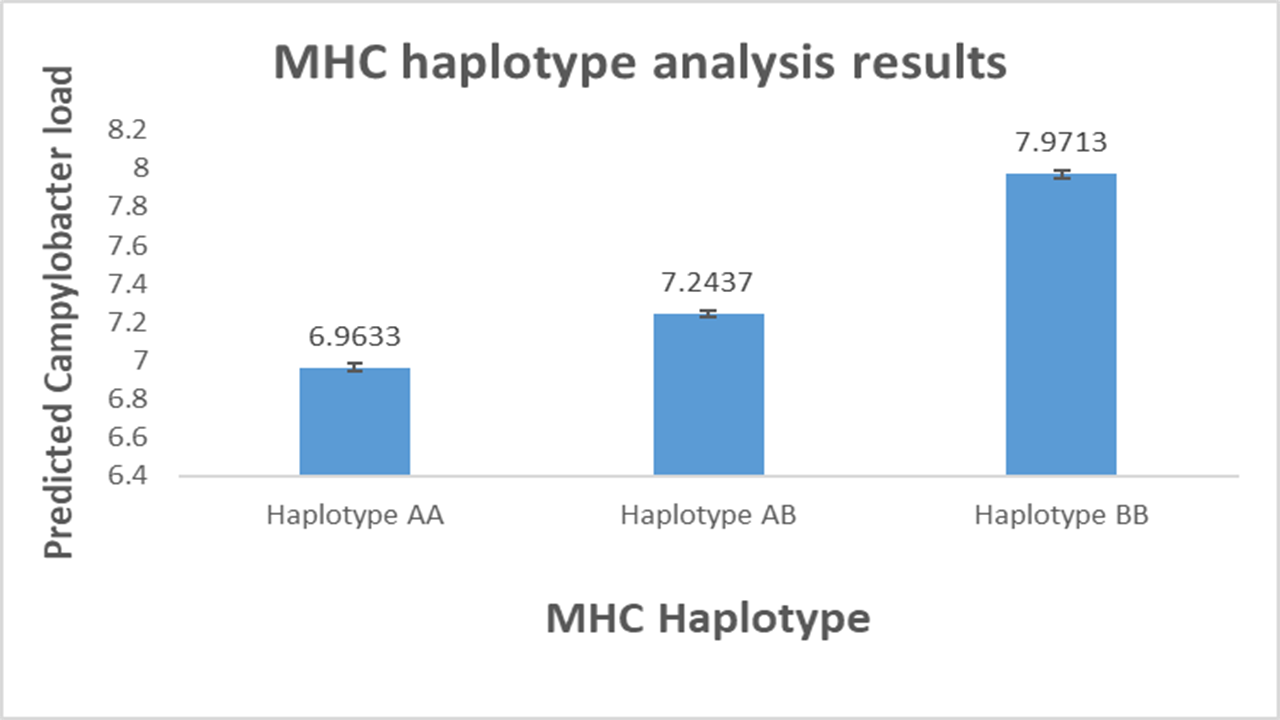

Supplement: Supplementary file 3 — Supplementary Figure S3. [file 41598_2020_79005_MOESM3_ESM.tif]

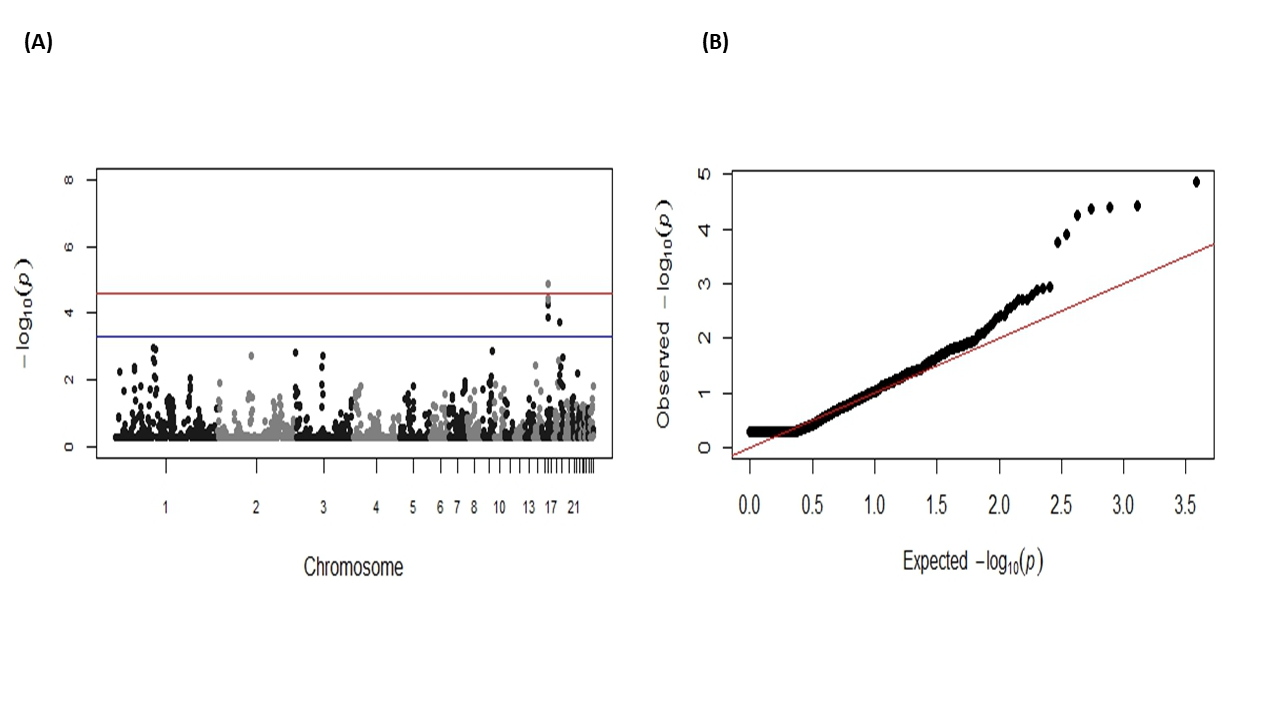

Supplement: Supplementary file 4 — Supplementary Figure S4. [file 41598_2020_79005_MOESM4_ESM.tif]

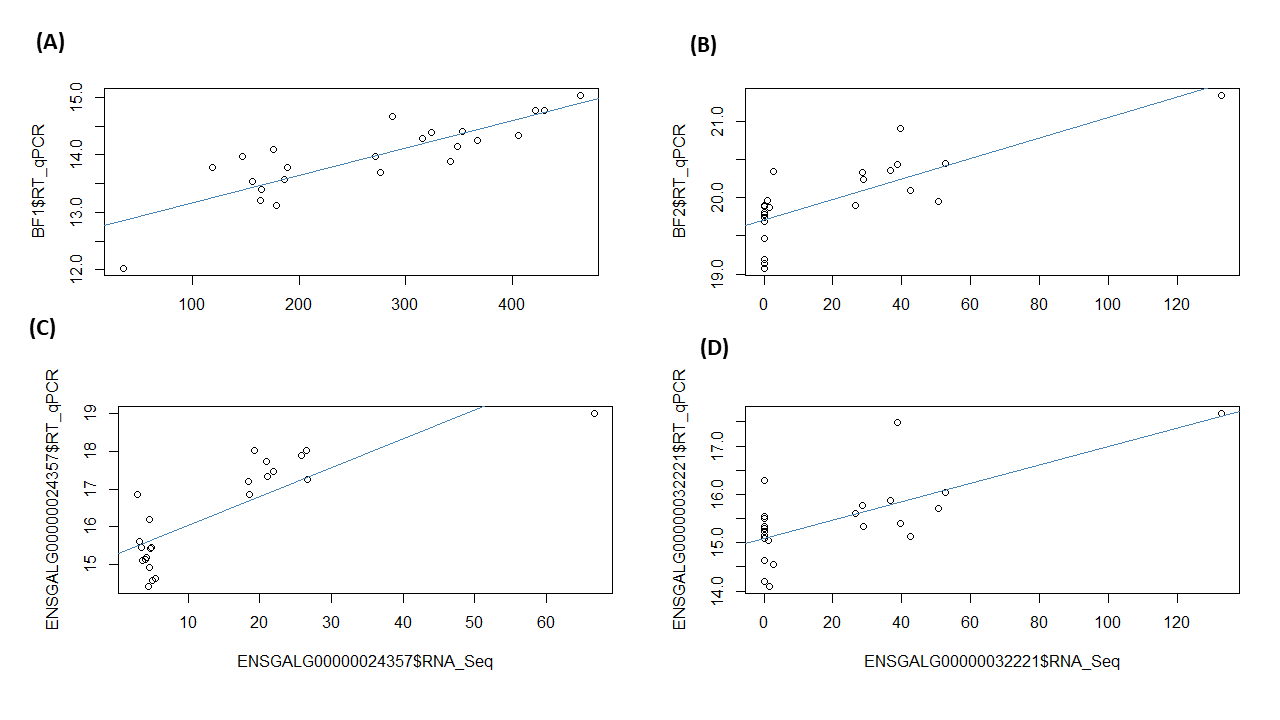

Supplement: Supplementary file 5 — Supplementary Figure S5. [file 41598_2020_79005_MOESM5_ESM.tif]
