# Supplementary material for: Quantitative trait loci and transcriptome signatures associated with avian heritable resistance to Campylobacter
Source: Sci Rep. 2021 Jan 12;11:1623. doi: 10.1038/s41598-020-79005-7 (PMC7804197; doi:10.1038/s41598-020-79005-7)
Supplement: Supplementary file 15 — Supplementary Captions. [file 41598_2020_79005_MOESM15_ESM.docx]

**Supporting** **Information**

**Supplementary Fig. S1.**  **Multidimensional scaling (MDS) analysis of the structure of the broiler population sampled**. MDS1 dimension (x-axis) is plotted against MDS2 dimension (y-axis).

**Supplementary Fig. S2. Patterns of linkage disequilibrium (LD) for SNP markers on chromosome 16 associated significantly with *Campylobacter* resistance in commercial chickens.** All the significant SNP markers were in high LD, illustrated with red colour, and were located within the same LD block (230kb) marked with triangle.

**Supplementary Fig. S3.**  **MHC haplotype analysis results**. *Campylobacter* caecal load (log-transformed CFU of *Campylobacter* per gram of caeca content) is plotted against the MHC haplotypes identified in the commercial chickens.

**Supplementary Fig. S4. Manhattan plot and Q-Q plot displaying the RHM results for chicken *Campylobacter* colonisation resistance.** (A) Genomic location is plotted against –log_10_(P) in the Manhattan plot. Genome-wide (*P* < 0.05) and suggestive genome-wide thresholds are shown as red and blue lines, respectively. (B) Q–Q plot of observed *P* values against the expected *P* values for *Campylobacter* caecal load (log-transformed CFU of *Campylobacter* per gram of caecal content).

**Supplementary Fig. S5. Correlation analysis results.** Scatterplots of expression levels quantified by qRT-PCR and RNA-Sequencing for *BF1* (A), *BF2* (B), *ENSGALG00000024357* (C) and *ENSGALG00000032221* (D) genes. Gene expression levels quantified by qRT-PCR (normalised Ct values) have been plotted against gene expression levels (TPM) quantified by RNA-Sequencing.

**Supplementary Table S1. List of SNP windows associated with *Campylobacter* colonisation resistance in Regional Heritability Mapping (RHM) analysis of the commercial chickens.**

**Supplementary Table S2. Variant Effect Predictor analysis results. Annotation of the SNP markers significantly associated with *Campylobacter* colonisation resistance identified by GWAS and RHM analysis in commercial chickens.**

**Supplementary Table S3. List of genes and non-coding RNAs located in the candidate regions for *Campylobacter* resistance identified by GWAS and RHM analysis in commercial chickens.**

**Supplementary Table S4. Details of the commercial chickens with low, average and high *Campylobacter* colonisation levels selected for RNA-Seq.**

**Supplementary Table S5. Expression (*cis*-) QTL analysis results.** List of SNPs identified by GWAS which were acting as *cis-* elements for genes located in the candidate regions for *Campylobacter* colonisation resistance.

**Supplementary Table S6. Expression (*trans*-) QTL analysis results.** List of SNPs identified by GWAS which were acting as *trans-* elements across the chicken genome.

**Supplementary Table S7. Allele specific expression (ASE) analysis results.** List of SNPs in the candidate regions for *Campylobacter* colonisation resistance which had significant ASE.

**Supplementary Table S8. Functional annotation clustering analysis of the genes located in the candidate regions for *Campylobacter* colonisation resistance in chickens.**

**Supplementary Table S9. Gene-specific primer sequences used in qRT-PCR to validate RNA-Seq results.** Primer sequences used to assess the expression levels of *BF2, BF1, ENSGALG00000032221* and *ENSGALG00000024357* genes in caeca tonsils of chickens with diverse levels of *Campylobacter load* by quantitative RT-PCR.
